# The human skin microbiome: factors affecting individuality and application in forensic investigations

**DOI:** 10.1007/s00414-025-03610-2

**Published:** 2025-10-31

**Authors:** Mishka Dass, Nathlee S. Abbai, Meenu Ghai

**Affiliations:** 1 Department of Genetics, School of Life Sciences, University of KwaZulu-Natal, Durban, South Africa; 2 School of Clinical Medicine, College of Health Sciences, University of KwaZulu-Natal, Durban, South Africa

**Keywords:** Skin microbiome, Factors affecting diversity, Forensic investigation, Individual identification, High-throughput sequencing

## Abstract

Differences in microbial communities have been observed across various skin sites, such as dry, moist, and sebaceous areas. These skin types influence the diversity of microbials present in each microenvironment. Commonly found skin microbes include *Staphylococcus epidermidis*, *Cutibacterium acnes* and *Corynebacterium* sp. Ethnicity, age, gender and health status are a few individual-specific factors that shape the skin microbiome. Every individual retains unique and distinct skin microbial communities despite constant exposure to environmental changes. In forensic investigations, human identification can be achieved through skin microbial trace analysis left behind on surfaces and objects. Temporal stability of the microbial profile, on skin, for up to two weeks, is an attractive feature for the implementation of skin microbiome analysis in forensic applications. Additionally, microbial traces can assist in determining geolocation and estimating postmortem interval. Although high-throughput sequencing technologies have accelerated microbiome research and provide species-level information, the skin is a low-biomass sample, and there are currently no standardised protocols from sample collection to analysis. Machine learning is rapidly advancing skin microbiome research by enabling the analysis of large and complex datasets to uncover patterns. These patterns can be used for predicting skin health conditions, matching skin samples to specific microenvironments, identifying individuals and inferring biogeographic origins. The present review highlights current research in the application of skin microbiome analysis for forensics and future potential applications for age and gender determination. Additionally, the factors affecting the skin microbiome diversity are discussed. Skin microbiome research will accelerate enrichment of microbiome databases, which could complement the standard STR typing in accurate human identification.

## Background

The skin is the largest organ in the human body and serves as a vital interface between the body and the external environment. The human skin can be categorized into 3 skin sites: dry, moist, and sebaceous [1]. Within these diverse physiological and topographical regions, the skin hosts a variety of bacteria, fungi, and viruses [2, 3]. It has been estimated that most (> 90%) of skin bacteria belong to: Actinobacteria (52%), Firmicutes (24%), Proteobacteria (16%) and Bacteroidetes (6%) [2]. At the genus level, in healthy individuals, human skin is dominated by one of three genera *Cutibacterium*,* Staphylococcus* and *Corynebacterium*, with community composition and diversity differing between individuals and skin sites [4]. Subsequent research has highlighted the individualized nature of the skin microbiome where unique microbial signatures and bacterial strains were observed among donors [5, 6]. Ethnicity, age, and gender are critical individual-specific factors that influence the skin microbiome. Additionally, host environment, health status and hygiene also affect the skin microbiome [7, 8]. In a study investigating the impact of cohabitation on the skin microbiome, ten sexually active couples were sampled across different skin sites (upper eyelids, outer nostrils, inner nostrils, armpits, torso, back, navel, inner thighs, bottom of feet, and palms of hands). It was found that cohabitation affected microbial community composition, but did not influence the skin microbiome as much as body location and individuality. Random Forest modelling was used to match individuals to their partners with 86% (*P* < 0.001) accuracy based on the feet swab data. It is plausible that microbiota on the feet of one partner matched to the feet of the other partner due to walking on shared surfaces and direct contact with each other, all of which likely leads to the sharing of microbiota. Other lifestyle factors influencing the skin microbiome include pet ownership, which increased microbial diversity and alcohol consumption which decreased microbial diversity [9]. Obesity also influences the skin microbiome, as Brandwein et al. reported a statistical correlation between body mass index (BMI) and the skin microbiome. Significant differences in alpha and beta diversity and community composition were observed among underweight, and obese individuals as well as normal and underweight individuals (*P* = 0.02). Additionally, the relative abundance of *Corynebacterium* was significantly correlated with BMI (*P* = 0.0002) and may serve as a potential marker for obesity and other metabolic syndromes [10].

The Human Microbiome Project (HMP) has paved the way for microbiome analysis since 2007. Developments in sequencing approaches such as metagenomic shotgun sequencing have allowed for the high-resolution characterization of the skin microbial community at strain and nucleotide level [3, 5, 11]. It has been established that even with exposure to the external environment, in adults, the skin microbiome is stable for two years [3] and microbial similarities observed within an individual outweighs the similarity between individuals [3, 5, 12, 13].

Skin bacteria are of interest to forensic science, due to its robustness compared to human DNA and the potential for individual identification, particularly when STR profiling is hindered due to extensively damaged, smudged, or partially recovered fingerprints and low-quality DNA samples [14]. Moreover, in cases involving mixed samples where human DNA fails to yield an accurate genetic profile, skin bacterial traces may help distinguish between individuals [15]. Skin microbial profiles have long-term stability in comparison to human DNA profiles [3, 5]. Temporal stability was demonstrated by Oh et al. and the skin microbial communities remained largely stable for two years despite constant environmental changes. Strain and single-nucleotide analyses revealed that stability was due to the persistence of strains during the two years rather than the reacquisition of common species from the environment. At sebaceous skin sites, bacteria and fungi were the most stable, while microbial communities at the foot site were the least stable [3]. Eukaryotic DNA viruses exhibited the most variation over time.

This review delves into the overview of the skin microbiome with regards to the factors that influence its microbial composition, and its application in forensics for individual identification, geolocation, and postmortem interval estimation.

## Methodology

An extensive literature search was conducted to identify relevant studies for inclusion in the review. The following databases were used to gather studies: Science Direct Scopus (https://www.scopus.com/) and PubMed (https://pubmed.ncbi.nlm.nih.gov/).

The following keywords were entered at the query step.

“application of skin microbes in forensics” OR “microbial traces for individual identification” OR “factors influencing the skin microbiome” OR “the human skin microbiome” OR “sequencing the skin microbiome for species level identification” OR “geolocation using skin microbes” OR “post-mortem estimation with skin microbes”

**Fig. 1:**
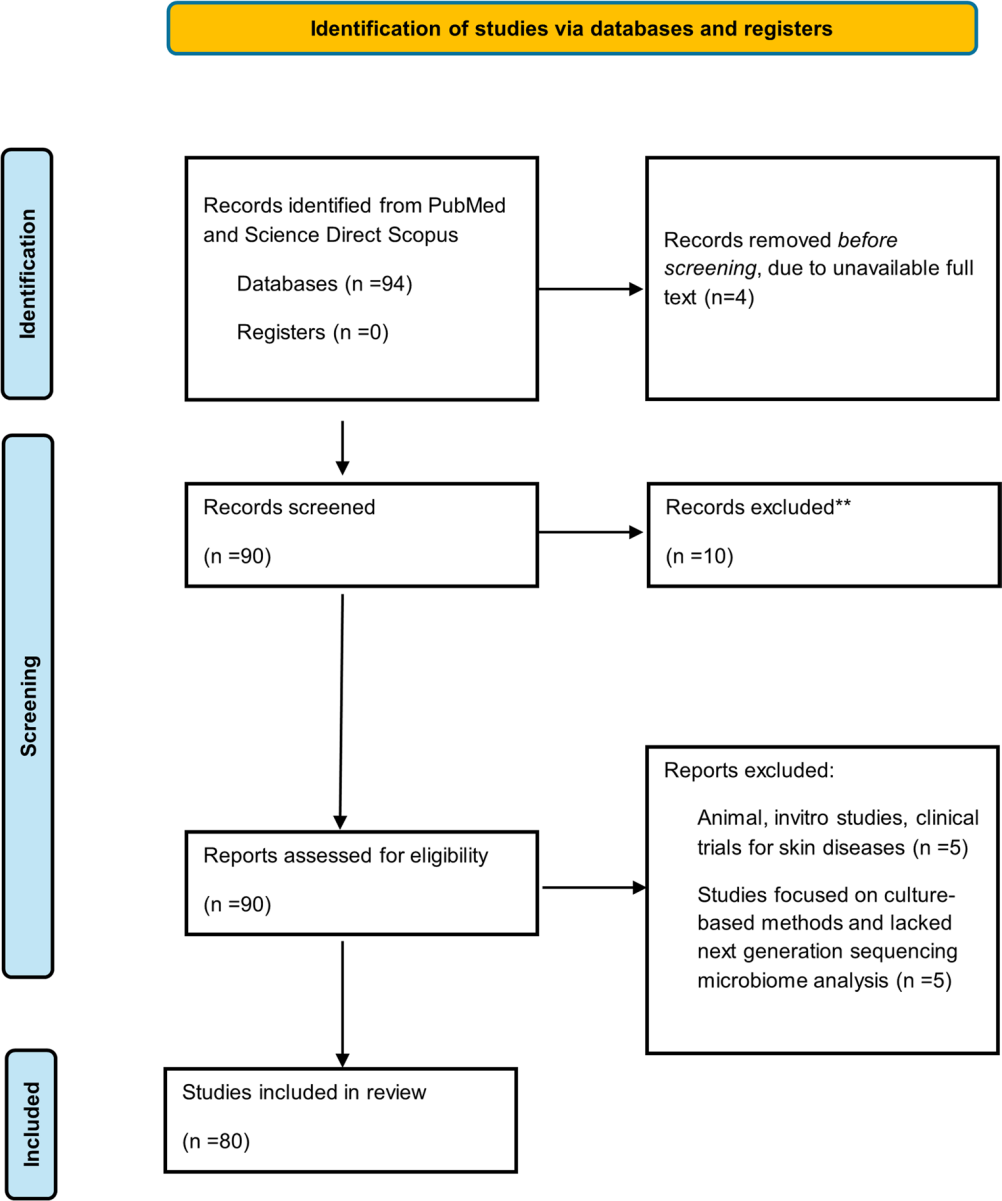
PRISMA flow diagram [16] describing the selection of studies for review of the human skin microbiome and its application in forensic investigations

At the screening phase studies were assessed based on the following criteria: Peer reviewed publications written in English and peer reviewed original research on healthy humans. Publications characterizing the skin microbiome by next generation sequencing, either full or partial * 16S rRNA* gene. Skin microbiome research that consisted of factors which affect microbial diversity (ethnicity, age, gender, hygiene, disease, environment). Forensic research investigating the touch microbiome, which is transferred to objects or surfaces, individual identification, postmortem interval estimation, geolocation and body fluid identification. Publications that included machine learning models for predictions based on microbiome data. Based on the above, a total of 90 studies were screen and 10 studies were excluded. After filtering and thorough assessment 80 studies were included in the review. A detailed flow diagram of the PRISMA workflow followed in this review is presented below in Fig. 1.

### Factors influencing skin microbiome diversity

#### Ethnicity

**Fig. 2:**
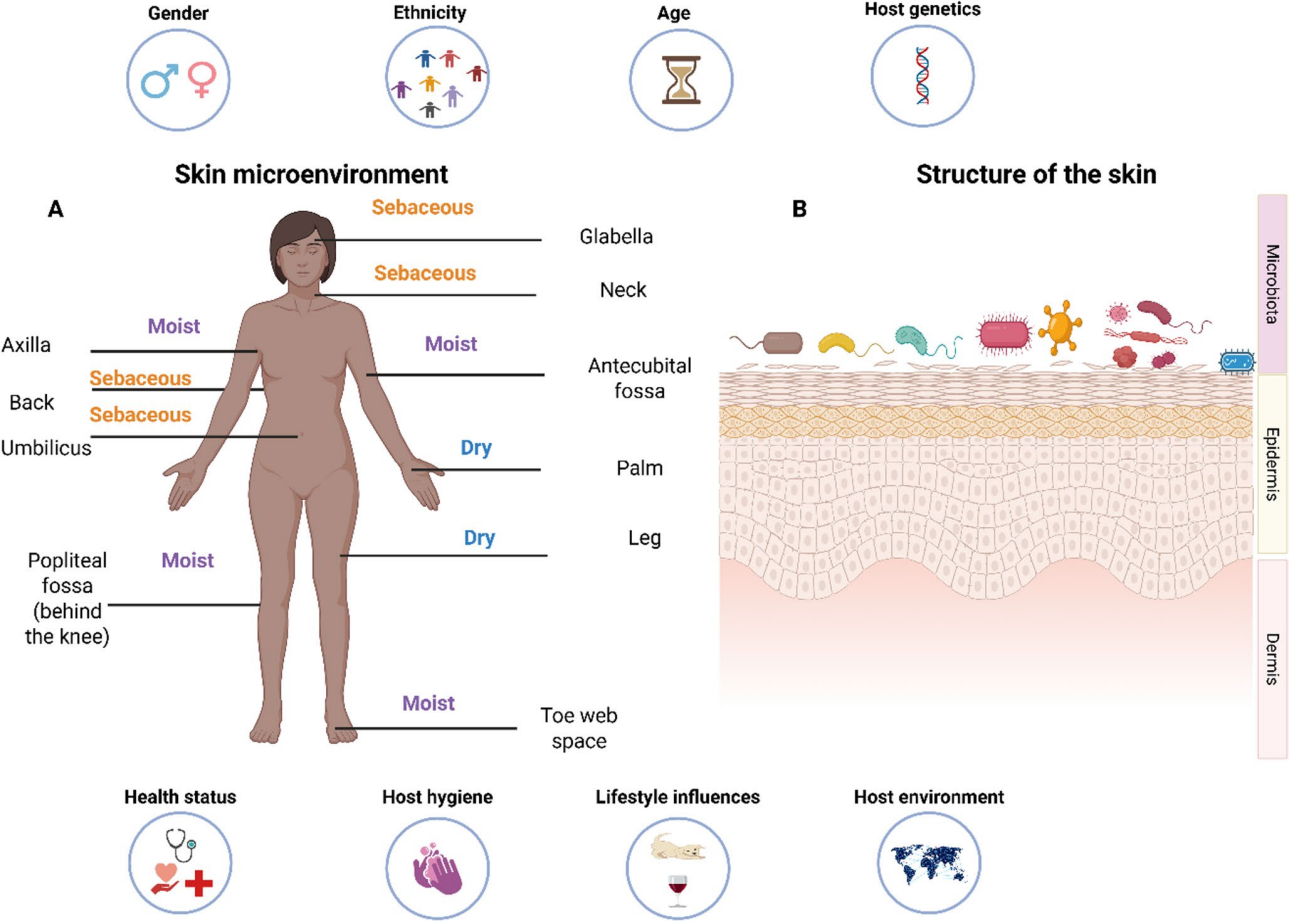
Skin microenvironment (**A**), structure (**B**) and factors which influence the microbiome (**C**). The structure of the skin is composed of the inner layer known as the dermis and the outermost layer known as the epidermis, on the epidermis, bacteria, viruses and fungi are found which make up the skin microbiome [1]. The microenvironment is represented by three sites: sebaceous, moist and dry[1, 8, 18] and the skin’s surface is characterized by acidity, high salt content, dryness and aerobic conditions [19]. The factors which influence the skin microbiome are depicted in circles [7]. (Created using Biorender.com)

Previous research has shown that ethnicity plays a role in the variation of the skin microbiome and is partially associated with lifestyle factors (Fig. 2). A study analysing the bacterial communities on the hands of women from Tanzania and the United States (US), found that bacterial communities were more similar within populations of the same country than between the two countries (*P* < 0.01). Firmicutes and Proteobacteria were the most abundant at the phylum level in Americans and Tanzanians respectively. At the family level, *Propionibacteriaceae*, *Staphylococcaceae* and *Streptococceaceae* were predominantly detected on American hand samples. These bacteria are typically associated with the hand microbiome. Interestingly, hand samples from Tanzanian participants presented a predominance of *Rhodobacteraceae* and *Nocardiodaceae.* Both these bacterial families are not the usual inhabitants of the skin but are more commonly found in soil and aquatic environments. This variation may be due to the influence one’s lifestyle and environmental exposure can have on shaping the composition of the skin microbiome, since Tanzanian participants had more exposure to the outdoor environment compared to US participants [17].

Perez et al. [20] analysed the forearm, axilla, and scalp microbiome of six ethnic groups (Caucasian- American, African American, African, Latin American, East Asian, and South Asian) residing in the United States. Differences at the phylum and genus levels were observed between participants of different ethnic origins across all skin sites. *Cutibacterium* was more abundant in the axilla and scalp than on the arm except in African and Latin Americans. While South Asian men had a lower abundance of *Acinetobacter* on the scalp and arm. East Asians had a higher abundance of the class *Alphaproteobacteria* and genus *Deinococcus*. Alpha-diversity measured by phylogenetic differences was higher in the arm than the scalp or axilla independent of ethnicity and was lowest in the axilla. Phylogenetic differences from the three skin sites in East Asians was significantly lower for other groups, including African American and Latin American in arm, African American, Caucasian American and Latin American in axilla, and African Continental and Caucasian American in scalp (*P* < 0.05).

The *ABCC11* gene and the SNP (R180) variant influences a reduction in secretion of metabolites from the axillary apocrine glands associated with odour formation. Dimorphic differences were observed between individuals with genotypes A/A and G/A or G/G, particularly between East Asian and European populations or African populations. Harker et al. [21] observed that the axillary microflora exhibited variations in abundance with a significantly increased abundance of *Staphylococcus* species (*P* = 0.0106) associated with genotypes G/G and G/A. While a reduction in the abundance of *Corynebacterium* species was observed in G/A and G/G genotypes of the *ABCC11* gene.

Analysis of metagenomic data from various studies focusing on the palms of hands revealed differences in the microbial composition of Chinese individuals compared to other ethnic groups [22]. Li et al. [23] observed a unique microbial composition in East Asians (*n* = 49) compared to Caucasians (*n* = 60) and Hispanics (*n* = 60). Total bacteria and Proteobacteria were significantly (*P* < 0.001) higher among East Asians than other groups. *Corynebacterium variabile* was only present in Hispanics while *Corynebacterium kroppenstedtii* was only detected in the East Asian group. Wang et al. [24] investigated the composition and diversity of skin microbiota on the forehead, cheek, jaw, opisthenar and palm of Chinese and Pakistani individuals living in the same environment. LefSe (Linear Discriminant Effect Size) analysis indicated Proteobacteria was more abundant in Pakistanis than in Chinese population. In contrast, Chinese samples had a higher abundance of Firmicutes. At the genus level, Pakistani samples were abundant in *Comamonadaceae*, *Curvibacter*, *Aquabacterium*. *Acinetobacter*, *Sphingomonas* and *Haemophilus*. While *Staphylococcus*, *Deinococcus* and *Prevotella* were more abundant in Chinese.

The above-mentioned studies indicate that skin microbiomes vary even within the same geographical location and country due to ethnicity and lifestyle [25].

The African Human Microbiome Portal (AHMP) was developed to gather metagenomic data from the African human microbiome body samples. To date, four research findings have contributed data on the skin microbiome of Africans [26]. Two of the studies focused on characterizing the skin microbiome of the Egyptian population, as well as comparing the composition, structure, and function of bacterial communities in patients with atopic dermatitis and healthy individuals [27, 28]. Ramadan et al. sampled the antecubital fossa and popliteal fossa of five Egyptians. Sequencing of the* 16S rRNA* revealed that the popliteal fossa had greater bacterial diversity than the antecubital fossa. *Klebsiella*, *Bacillus*, *Pseudomonas*, *Escherichia*, *Meiothermus* and *Corynebacterium* were the dominant genera present among all participants at both sample locations [27]. The overall results of the study indicated that environmental factors mould the composition of the skin microbiome in a particular geographic region. Subsequent research by Ramadan et al. showed that bacterial diversity among subjects with atopic dermatitis displayed lower bacterial diversity than healthy subjects. At the genus level, subjects showed significant abundance variations by disease severity, age, locality, or immune response. Among these genera, *Streptococcus*, *Cutibacterium* and *Corynebacterium* were identified as specific signatures for atopic dermatitis in children, adolescents and adults. *Staphylococcus* was identified as a potential biomarker for atopic dermatitis [28]. The penile skin microbiome was evaluated among 238 black South African men with the Human Papillomavirus. *Corynebacterium*, and *Prevotella* were identified as the most abundant genera among these men [29]. A study performed in Uganda aimed to evaluate the relationship between the penile microbiota of uncircumcised men and the bacterial vaginosis (BV) status of their female partners. The researchers hypothesized that in uncircumcised men characterized as community state types (CSTs) 1–3, microbiota was of low bacterial density and predominantly comprised of skin-associated bacteria such as *Corynebacterium* and *Staphylococcus*. These bacteria were significantly more prevalent in CST 1–3 compared to CST 4–7 (*P* < 0.001). Additionally, the findings were associated with men whose female partners had a normal Nugent score (i.e., without BV). In contrast, men whose female partners had BV showed a significantly higher prevalence of BV-associated bacteria: *Prevotella*, *Porphyromonas*, unclassified *Clostridiales*, *Dialister* and *Mobiluncus* in CST 4–7 compared to CST 1–3 (*P* < 0.05). The results indicate a microbial exchange of BV-associated bacteria through sexual intercourse and may explain the prolonged duration and recurrence of BV. Since uncircumcised male partners can be carriers of BV associated bacteria, managing male carriers could reduce the likelihood of ongoing infection and repeated transmission in women [30].

#### Gender

Gender plays a role in the diversity of the skin microbiome (Fig. 2) as differences in species composition have been noted between male and female counterparts [23, 31, 32]. These differences are likely due to sex- specific properties of the skin such as thickness of skin, hair growth, sweat, skin pH, hormones, and sebaceous gland production [33]. Using pyrosequencing of the * 16S rRNA* gene, Fierer et al. showed that significantly different (*P* < 0.001) bacterial communities are present on the palms of men and women, and shared taxa were more abundant on the palms of one gender than the other. The following genus showed differential abundance in males and females: *Cutibacterium* formerly known as *Propionibacterium* (37% more abundant on men), *Corynebacterium* (80% more abundant on men), *Enterobacteriales* (400% more abundant on women), *Moraxellaceae* (180% more abundant on women), *Lactobacillaceae* (340% more abundant on women), and the *Pseudomonadaceae* (180% more abundant on women). Overall, the palms of women had higher bacterial diversity than men [32]. Similarly, a study employing *16S rRNA* gene sequencing to investigate the facial microbiome found that bacterial diversity at the genus level was greater in women than in men. LeFSe analysis at the genus level indicated significant differences (LDA score| >2.0, *P* < 0.05) between genders for 15/22 genera. At the phylum level, the relative abundance of Actinobacteria was similar between genders, however, the next most abundant bacteria were Firmicutes in males and Proteobacteria in females [31]. The influence of gender, age, and race/ethnicity on the human axillary (underarm) microbiome was investigated by Li et al. [23] and IS Pro analysis of the *16S–23S* rRNA intergenic spacer region revealed a differing composition of *Corynebacteria* among males and females. *Corynebacterium amycolatum* and *Corynebacterium kroppenstedtti* were only detected in males while *Corynebacterium urealyticum* and *Corynebacterium variabile* was only present in females.

From a forensic perspective, Richardson et al. [34] tested whether prediction of gender was possible by identifying differentially enriched taxa among samples using DESeq2. Samples were collected from 37 participants and 28 dorm rooms. Swabs were taken from the dominant hands of participants, desks, floors, bed sheets, and doorknobs. Common surfaces swabbed included dormitory and bathroom door handles, elevator buttons, and hallway floors and sampling was done at four time points for 3 months. *Lactobacillus iners* and *Corynebacteria* species were enriched in women and men respectively. These abundant taxa were used in a random forest model to predict gender. The model had an accuracy of 80% and an error ratio of 2.5. The presence of roommates linearly correlated with an increase in classification error of 18%. This is due to the microbial exchange that occurs between individuals, causing a reduction in bacterial abundances and an increase in model error. These findings highlight the importance of considering the presence of cohabitating individuals and the mixing of bacterial signatures that can mask the true identity of individuals present in a particular environment.

#### Age

Chronological age determination using skin microbials may have benefits in forensic investigations to estimate the age profile of unidentified individuals or suspects based on the diversity of microbial communities. However, detailed research needs to be conducted for its applicability to real-world scenarios, even if machine learning algorithms can assist in highly accurate predictions. Age-related skin changes result from internal factors (genetics and gender), environmental factors (pollution, exposure to the sun and climate), and lifestyle factors (exercise, stress, sleep, nutrition and skincare routine) (Fig. 2). These changes reduce sebum and sweat production, alter lipid composition, decrease collagen and elastin, and lead to skin dryness, impacting the skin’s physiology and microbiome [35]. The skin microbiome is formed rapidly after birth when a baby’s skin is colonized by the mother’s microbiome. Thereafter, initial microbes are outcompeted by new strains of bacteria due to environmental exposure and host maturation [36]. Interestingly, Huang et al. [37] demonstrated that the skin microbiome is a more accurate predictor of chronological age than oral and gut microbiomes, with an accuracy of four years within chronological age. Howard et al. [38] investigated the influence of age on the skin microbiome by performing * 16S rRNA* pyrosequencing analysis. The study enrolled 158 Caucasian women aged 20–24, 30‒34, 40‒44, 50‒54, 60‒64, and 70‒74 years. Swabs were taken from the buttocks, facial skin, and forearm. In addition to sequencing, host factors were also analysed such as the sebocyte gland area, skin lipids, natural moisturizing factors and antimicrobial peptide measurements. Overall analysis indicated that bacterial diversity increased across all skin sites as women aged. Facial sebum production decreased among women over 40 years old and this positively correlated with *Cutibacterium* and negatively correlated with *Streptococcus*, *Acinetobacter*, *Corynebacterium*, and *Methylobacterium*‒*Methylorubrum*. Sebum lipids act as a food source for *Cutibacterium*, likely accounting for its decrease in abundance [39]. The inverse correlation between other bacteria and the sebaceous gland may be due to the reduced presence of *Cutibacterium* (the most abundant facial skin bacteria), which allows for the colonization of the niche by other bacteria. In addition, the loss of antibacterial components such as Sapienic acid (C16_1) in sebum could have played a role, which decreased with age at all three body sites. Sphingolipids were elevated with increased age, mainly at the forearm and buttocks and may be responsible for reducing the abundance of *Lactobacillus*. Correlation analysis of bacterial genera with an average relative abundance greater than 1% across all three body sites combined showed that *Cutibacterium* and *Staphylococcus* were negatively correlated with an increase in total natural moisturizing factors. In contrast, *Corynebacterium*, *Micrococcus*, *Streptococcus*, *Anaerococcus*, *Finegoldia*, *Methylobacterium‒Methylorubrum*, *Acinetobacter*, *Enhydrobacter*, and *Leptospira* were positively correlated with these factors [38]. This study highlighted age-associated changes in the skin microbiome in relation with host factors.

Li et al. [23] explored the influence of age on the axillary microbiome. Skin swabs were collected from the underarms of 169 healthy individuals aged 18–30, 35–50 and 55 + years. Results indicated that total bacteria were higher in older participants (55 + years of age) in comparison to middle age (35–50 years old; *P* = 0.068) and younger participants (18–30 years of age; *P* = 0.037). Total bacterial levels were similar among middle-aged and younger participants. The diversity of *Corynebacteria* species increased with age as for older participants, ten *Corynebacteria* species were detected, while seven and eight species were detected in middle-aged and younger participants, respectively. The study provided evidence that the axillary microbiome is influenced by age, particularly with respect to *Corynebacteria* species. The body malodour caused by *Corynebacteria species* may contribute to the odour often associated with elderly individuals. However, further research is required to verify if increased diversity of *Corynebacteria* is linked to different types of axillary metabolites that may contribute to bad odour in seniors.

Myers et al. [40] conducted a recent multi-study analysis looking at cheek microbiome datasets made up of * 16S rRNA* amplicon sequencing data and paired clinical data, to establish microbial taxa with a potential association with skin quality or signs of aging. Subjects from the study were aged between 18 and 69 years old. A negative relationship between microbial diversity and transepidermal water loss (TEWL) was found. While there was a positive relationship between microbial data and age, where older individuals showed higher microbial diversity than younger individuals. In addition, a positive correlation between microbial diversity and Crow’s feet wrinkles was found (GCFW). The authors also uncovered microbial taxa that may be associated with wrinkles, TEWL and corneometer measures. Cheek samples with fewer wrinkles were associated with the following commensal genera: *Staphylococcus*, *Kocuria*, *Peptrostreptococcus* and *Lysobacter*. While individuals with higher grades of wrinkles possessed environmental-related bacteria such as *Brevibacterium* and *Kaistella*, which are often associated with inflammatory skin conditions. *Staphylococcus* and *Bacillus* were related to low TEWL and high corneometer measures were associated with *Janibacter*, *Rosemonas*, *Sphingomonas* and *Lactobacillus*. Although *Cutibacterium* was the most abundant genus in the dataset and has been shown in the literature to be an abundant bacterium on the cheek, there was no significant relationship with age. This could be due to the inclusion of age skin parameters not typically associated with *Cutibacterium* presence. Overall, the skin microbiome was influenced by age, demonstrating clear differences in diversity between older and younger individuals, with the elderly displaying increased microbial diversity.

#### Skin microbiome: forensic applications

Forensic applications of skin microbiome that have been widely documented include individual identification, postmortem interval estimation and geolocation are elaborated below.

#### Individual identification

**Fig. 3:**
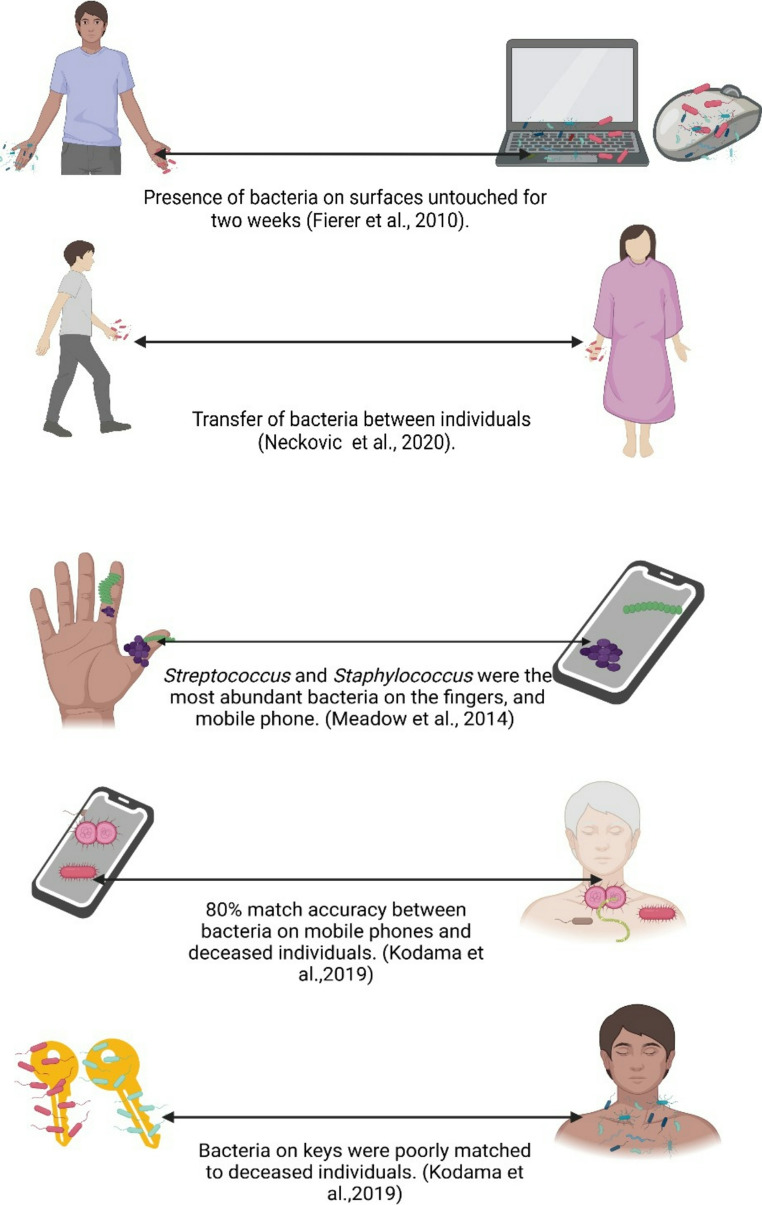
Transfer of skin microbes on frequently contacted objects and between persons (Created using Biorender.com)

Locard’s exchange principle, which states “every contact leaves a trace” [41] can be applied to microbial forensics for individual identification (Fig. 3). This can be achieved by detecting unique skin microbial signatures left on objects touched by an individual, resulting in a transfer of bacterial DNA between surfaces or objects touched and the person who deposited the traces [14, 42–44].

The following studies have demonstrated the potential of the skin microbiome to identify individuals. Fierer et al. [45] conducted a range of experiments to prove that microbial communities from the surface of objects such as computer mouse/keyboards can be matched to a donor’s skin profile. Barcoded pyrosequencing of the * 16S rRNA* gene was used to determine the composition of bacterial communities and results showed that the bacterial community on the fingertips of donors were similar to the bacterial communities present on the donor’s keyboard. Additionally, bacterial communities from donors’ and their keyboards were more similar to each other compared to the bacterial communities from the fingertips and keyboards of different donors. In the next experiment, authors investigated whether the bacteria on a personal object were more similar to bacteria found on the donor’s skin than the general population. The palms and computer mouse of donors who had not touched their equipment for more than 12 h were sampled. Phylogenetic distance was calculated between the bacterial communities on each mouse and donor’s palms, comparing this distance to distances between mouse bacterial communities and the bacterial communities of 270 palms from the database representing the general population. Results showed that the bacterial community on each mouse was more similar to the bacterial community on the donor’s palms than the other palms in the database, irrespective of the distance metric used. Temporal stability of bacterial communities from skin swabs was also evaluated, which revealed that even after exposure to the indoor environment, there was little difference observed in bacterial communities for up to two weeks [45].

Similarly, in a study by Wilkins et al. [46], skin (*n* = 380) and household surface (*n* = 288) bacteria were collected during four seasons to assess the accuracy of matching individuals to their household surface over extended periods. The study revealed a decrease in accuracy with increasing time intervals between skin and household surface samples, with most operational taxonomic units (OTUs) persisting less than one season. Subsequent research by the same group, indicated that diurnal variations in the human skin microbiome impacted accuracy. This study observed significant changes (*P* = 0.008) in relative abundance of 160 species between morning and evening at all sampling sites. The findings indicate the importance of considering the temporal decay of bacterial traces and diurnal bacterial patterns in developing the skin microbiome as a potential tool for identifying individuals [47].

Mobile phones are extremely popular amongst the majority of the world’s population and are frequently touched by their owners. Therefore, it was hypothesized by Meadow et al.[48] that mobile phones could be used as “personal microbiome sensors”. This hypothesis was tested by collecting 51 swabs from the phone’s surface as well as from the owner’s index finger and thumb. The most abundant operational taxonomic units (OTUs) across phones, fingers and genders were *Streptococcus* and *Staphylococcus*. Bacterial communities from mobile phones had a greater similarity to the owners than other individuals and 22% of bacterial taxa from owners were recovered on mobile phones compared to 17% shared with the phones of other individuals. Furthermore, when the operational taxonomic units (OTUs) of each participant were compared, it was found that participants shared 5% more OTUs with their own phone compared with other participants. While 82% of OTUs were common between index fingers and phones, 96% of OTUs were shared between thumbs and index fingers. Thus, the matching of mobile phones to their owners has potential that needs to be assessed further with a larger sample size for forensic application.

Neckovic et al. [49] determined the possible transfer of the skin microbiome between individuals and onto substrates and vice versa. Cotton fabric, paper, glass marbles and hands were analysed for transfer of bacteria. Assessment of the mode of transfer was carried out by dividing the six study participants into three pairs and each pair engaged in two transfer modes. In transfer mode 1, participants shook hands and then rubbed a substrate with their right hand. Transfer mode 2 involved individuals rubbing a substrate with their left hand, exchanging the substrate with their partner, and then rubbing the exchanged substrate with their left hand. Microbial DNA was extracted from participants and substrates, followed by * 16S rRNA* sequencing. Results from PERMANOVA showed significant differences (*P* < 0.001) in the Jaccard distances between each participant pair. This demonstrates the potential to distinguish between skin microbiomes among individuals and highlights the clustering effect observed within participant pairs due to the likely transfer of hand bacteria between individuals. The study confirmed the occurrence of human skin microbiome transfer between all participant pairs, irrespective of the substrate type or transfer mode.

The “touch microbiome” was evaluated in a pilot study by Procopio et al. [42]. Skin swab samples were collected from the dominant hand of 11 Italian volunteers. To simulate real-life forensic crime scenes, volunteers also deposited their fingerprints on non-sterile glass slides, which were then exposed to the environment for 30 days. Sequencing region V4 of the * 16S rRNA* gene enabled the successful generation of 20/22 touch microbiome profiles in comparison to 5/22 STR profiles identified using human DNA obtained from skin swabs and glass slides. Overall, the bacterial composition differed between skin swabs and glass slides, as fewer bacterial taxa were detected on glass slides, indicating that transferred bacterial fingerprints do not fully represent the skin microbiome. The skin core microbiome (SCM) was defined by the authors as taxa always present in all skin samples, which included six amplicon sequence variants (ASV), three of which were resolved at species level: *Streptococcus agalactiae*, *Actinobacillus delphinicola*, and *Anaerosinus glycerini*, two at order level: *Bacillales* and *Actinomycetales*, and one at family level: *Enterobacteriaceae*. Unique bacteria such as *Pseudoalteromonas ruthenica*,* Macrococcus brunensis*,* Corynebacterium renale* and *Cytophaga xylanolytica* were also detected for the first time in this study and may have the potential to possibly identify individuals when multiple suspects have been identified and their skin microbiomes swabbed. While promising results were observed, the authors suggested that future studies with a larger sample size, increased datasets, more time intervals for bacterial stability evaluation, and increased surface examination are needed to confirm the applicability of the “touch microbiome” for forensic investigations.

A florescent multiplex system comprising of seven microbial markers was developed by Yao et al. [50], to distinguish between skin, saliva and faeces. The authors opted for a non-sequencing approach due to the cost efficacy and simplicity of applying PCR analysis to the forensic workflow. Skin samples were collected from six different locations (palm, antecubital crease, inguinal crease, cheek, upper back and toe web space). *Finegoldia magna*, *Corynebacterium tuberculostearicum* and *Cutibacterium acnes* were selected as skin-specific microbial markers and were not detected in saliva or faeces. Beta diversity analysis showed clear clustering among skin, saliva and faeces. Furthermore, a machine learning algorithm was developed based on random forest regression to classify skin samples into four different microenvironments (oily, moist, dry, foot). The model was able to predict the skin microenvironment of the 492 skin samples with 79% accuracy. Overall, the results of this study can be used towards the advancement of body fluid/tissue identification in forensics. Additional research could be done to see if additional microbial markers can be included for improved accuracy, furthermore, mock forensic samples simulating real-life crime scene evidence should be evaluated on the system to determine the effects of the environment on the sample.

SMRT sequencing was performed to sequence the full length of the * 16S rRNA* gene to determine its application in microbial forensics by analyzing the microbiota present in palmar skin, oral mucosa and nasal cavity. From the respective body sites, 167 swabs were collected from 19 male Han participants and distinct bacterial biomarkers were discovered. Bacterial biomarkers of the palmar skin included *Cutibacterium acnes*, *Staphylococcus caprae*, *Pseudomonas aeruginosa*, *Staphylococcus hominis* and *Neisseria flavescens*. Three classification models were developed to predict body site origin of biological samples based on bacterial species. The Random Forest and XGBoost models were similar in performance and had a classification accuracy of 97%. In contrast, the KNN model had a lower classification accuracy of 79%, performing less effectively than the other two models. It was interesting to note that the bacterial community in the palmar skin was more balanced than that of the oral mucosa and nasal cavity [51]. This distribution pattern may be attributed to the prolonged exposure of the palmar skin to the external environment and surfaces. This allows for the coexistence of a larger diversity of bacteria and limits the relative abundance of individual bacterial genera [52].

Additional studies on the application of the skin microbiome for individual identification and transfer potential are listed in Table 1.

**Table 1:** Overview of the forensic applications of the skin microbiome for individual identification, geolocation inference and postmortem interval estimation

Forensic Application	Location/site and sample size	Sequencing method/PCR technique	Machine Learning or Statistical method applied	Main findings	References
Individual identification	Mobile phone *N* = 91	* 16S rRNA* gene sequencing, V4 region	Supervised learning- random forest model	Bacteria of an individual matched to their phones.	Lax et al. 2015 [67]
	Swabs from the foot, hand and manubrium. *N* = 72	Targeted sequencing (hidSkinPlex clade specific markers-Illumina platform).	Supervised learning- regularized multinomial logistic regression and 1-nearest-neighbor classification.	hidSkinPlex consisted of 286 markers. Accurate classification of foot (92%), manubrium (96%) and hand (100%).	Schmedes et al. 2018 [68]
	Forehead swabs *N* = 66 (swabs) *N* = 11 (participants)	* 16S rRNA* amplicon sequencing	Canberra distance	Personal identification achieved at 95%. Overtime accuracy was 85% during first- and second-year sampling. From 89 public datasets, accuracy was 78%.	Watanabe et al. 2018 [69]
	Swabs from palm, navel, plantar and groin for skin site recognition in sexual crimes. *N* = 14	* 16S rRNA *sequencing, V3-V4	Random forest algorithm	Predictive model accurately distinguished the four skin sites.Skin was distinguishable from saliva and vaginal fluid.	Huang et al. 2024 [70]
	Hands and computer keyboards of individuals. *N* = 10	Ribotyping of the * 16S, 23S rRNA *genes and variable number tandem repeat (VNTR) of* S. epidermis.*	Unifrac metric and Bray Curtis	Similarity rates between the skin and environmental bacteria ranged from 31.74%−77.14%.	Yılmaz et al. 2024 [71]
	Hands and knives were touched by individuals. *N* = 5 (hands) *N* = 5 (knives)	* 16S rRNA* sequencing V1-V9 region	Weighted Unifrac distance	Unifrac distance showed no similarity between hands and post-transfer knife handle sample.8.28% of individual specific microbials were detected after 4 h and 6.95% remained after 24 h.	Karadayı et al. 2024 [15]
Postmortem interval estimation	Swabs from the ear and nose of a human cadaver. *N* = 144 (swabs) *N* = 21 (cadavers)	*16S rRNA *sequencing, V3-V4 region.	*k*-nearest- neighbor regression model	Genus and family were more informative taxonomic levels than species. PMI estimation was ± 2 days.	Johnson et al. 2016 [53]
Geolocation	*N* = 104 (phone) *N* = 211 (shoe)	* 16S rRNA* gene sequencing, V4 region	Supervised learning- random forest model	Bayesian method matched shoes to participants based on floor similarity. Shoe and phone bacteria clustered by location, indicating shared low abundance microbes among individuals in the same space.	Lax et al. 2015 [67]

#### Postmortem interval

**Fig. 4:**
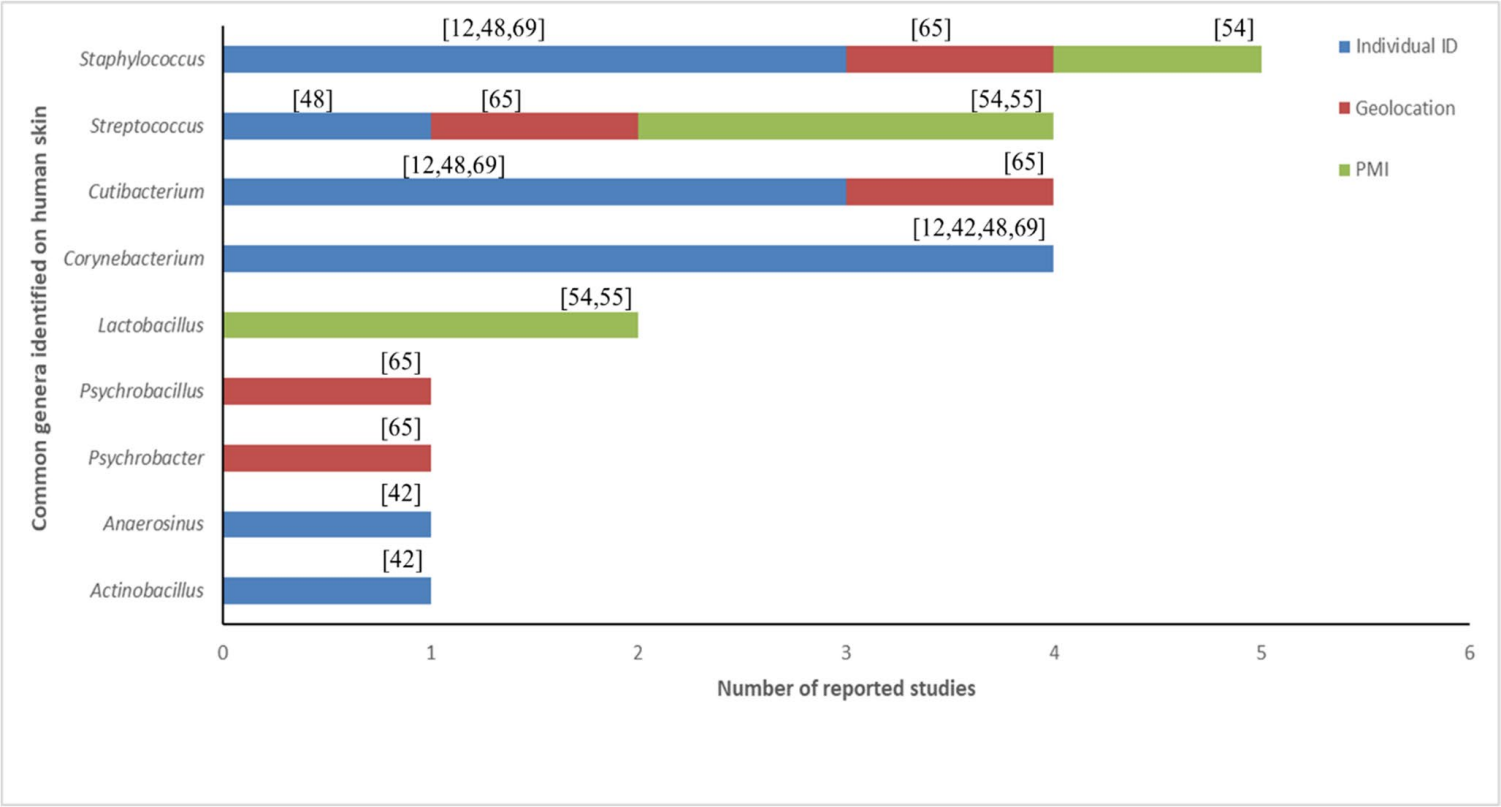
Genera identified on the skin in different forensic studies investigating individual identification, geolocation and postmortem interval estimation

The skin microbiome could also have implications for determining postmortem interval (PMI) and geographical locations (Fig. 4; Table 1). With regards to PMI, Johnson et al. [53] developed a statistical regression algorithm to predict PMI from skin bacteria derived from the nasal and ear canals of decomposing human cadavers. Results also showed that genus and family rather than species provided the most informative taxonomic levels. The most informative microbes for the prediction of accumulated degree days (ADD) consisted of the following genera: *Caulobacter*, *Acidisphaera*, *Phenylobacterium*, *Dactylosporangium*, *Nocardioides*, *Staphylococcus*, *Peptoniphilus*, *Vagococcus*, *Pseudonocardia*, *Oenococcus*, *Macrococcus*, *Symploca*, *Pelomonas*. The model had a low error rate and a PMI estimation of ± 2 days. This was a vast improvement compared to current PMI techniques such as forensic entomology. Kodama and colleagues [54] attempted to determine the skin microbiome from 16 scenes where death had occurred in Honolulu and investigated whether objects present at crime scenes could be matched to the deceased individuals. Results indicated that the postmortem skin microbiomes remained stable for up to 60 h postmortem and were similar to microbiomes of antemortem populations. Objects could be linked to deceased individuals 75% of the time; smoking and medical devices were matched to the deceased with 100% accuracy. However, car and house keys were poorly matched to individuals, whereas phones were matched to the deceased with 80% accuracy.

Iancu et al. [55] obtained similar microbial stability results to Kodama et al. when skin microbiome samples were collected from the faces and hands of eight cadavers within immediate arrival at a morgue (T0) and 12 h later (T1). The taxonomic characteristics of the skin microbiota varied across different body sites, with no differences in taxonomic profiles between (T0 and T1). The most abundant phyla observed in both sampling periods were *Firmicutes* (T0: 61.6%, T1: 57.4%), followed by *Actinobacteria* (T0: 20.1%, T1: 18.4%), *Proteobacteri*a (T0: 10.9%, T1: 15.2%), and *Bacteroidetes* (T0: 4.0%, T1: 5.2%). These dominant phyla comprised 87.6% of the skin microbiota composition at T0 and 96.2% at T1. *Staphylococcus* was dominant at the genus level and was most abundant at T0 (30.8%) compared to T1 (19.8%). *Streptococcus* (T1: 12%, T0: 90.2%), *Lactobacillus* (T1: 7.6%, T0: 6.6%) and *Clostridium* (T1: 5.4%, T0: 4.4%) were also prevalent genera among samples. The study also revealed that specific microbial signatures could be linked to causes of death such as cardiovascular disease, while high blood alcohol levels were associated with reduced bacterial richness and diversity. Additionally, the locations where the bodies were found appeared to influence microbiome diversity. Overall, the skin microbiome seems promising for enhancing the estimation of PMI in a non- invasive manner and can be applied alongside other forensics methods for a robust determination of PMI. Further studies will need to work on evaluating the relationships between chronological age, the number of diseases, the number of medications used and microbiome diversity as this will provide an array of additional information for determining PMI [55].

In addition to PMI estimations, the postmortem microbiome may have other potential uses. For example, manor of death (MOD), may also be worth exploring in the future as demonstrated by Mikles et al. [56] whereby pediatric deaths (≤ two years of age) were investigated to determine the postmortem microbiome. In this cross-sectional study of 53 pediatric autopsy cases using * 16S rRNA* sequencing, the authors aimed to characterize biomarkers for predicting MOD. The autopsy cases included males and females of black and white race groups with age ranging from 0 days old to 2.22 years. The following body sites were swabbed totalling 298: ears, eyes, nose, mouth, umbilicus, rectum, trabecular space, interhemispheric fissure, cardiac blood. It was found that sex, race, age, body site, and MOD all had significant impacts on microbiome composition, with notable interactions observed among MOD, race, and age. Significant bacterial taxa were identified in relation to gender and race. Taxa most strongly associated with racial differences was *Leptotrichiaceae* (*p* = 0.0098), *Sneathia* (*p* = 0.006), and an uncultured *Sneathia* species (*p* = 0.001). These were predominantly found in white individuals and appeared to be most strongly associated with male sex and non-accidental death. Lower prevalence of *Sneathia* was also observed in black, male, non-accidental death cases. LEfSe analysis based on gender identified *Escherichia-Shigella* (*p* = 0.010) as the sole significant taxon, which was associated with females and white individuals who died of accidental causes. Although there is an overlap between MOD, gender, race and the presence of the bacteria *Sneathia*, it is still unclear how this bacterium becomes part of the infant’s microbiome. It cannot be assumed that *Sneathia* is linked to MOD. More detailed studies are required to detect specific biomarkers that relate to circumstances surrounding death.

#### Geolocation

Microbes have the potential to aid in geolocation by distinguishing between primary and secondary crime scenes, locating clandestine graves and identifying suspects. This is possible due to the varying composition and function of microbial communities based on geographical location, climate (including precipitation rates, altitude and temperature), soil properties and the host properties or energy sources present in the environment (Fig. 4, Table 1) [57]. The studies below show the effect of geographical location on gut, saliva, skin and environmental microbiome.

#### Gut Microbiome

The analysis of gut samples from 2164 Chinese participants from 15 provinces, showed that different geographical locations influenced the diversity of gut microbiomes. The geographical location (province or megacity) of participants accounted for the highest proportion of microbial variance (17.9%) whereas host factors such as occupation, sanitation, urban/rural status and diet contributed to less than 1% of variance [58]. In another study focusing on the Chinese population, gut microbial community variation was strongly associated with the host’s location [59].

#### Urban microbial ecosystem

Danko et al. [60] determined the urban microbiome and antimicrobial resistance genes by analysing 4 728 metagenomic samples from mass-transit systems in 60 cities for three years. These samples were collected from frequently contacted surfaces such as transit stations, ticket kiosks, turnstiles, seats, railings and benches. If a city did not have a subway system, then the most common mode of transport was investigated. Each city represented distinct microbial profiles that were influenced by climate and geographic differences as well as a “core” urban microbiome. These results indicated that region-specific source tracking is possible and, in a forensic context may help reconstruct timelines of events involving the movement of a suspect or victim or provide information about the location of a specific crime.

In a more recent study, Zhang et al. [61] developed a machine learning tool known as microbiome geographic population structure (mGPS) to enable the tracing of samples to their water body, country or city of origin by microbial relative sequence abundances. The study analyzed 4 070 samples from 40 cities, 231 samples from 13 countries and 131 samples from 9 water bodies. These datasets included collections from mass transit systems, soil and marine environments. Results revealed that mGPS was able to predict the source city for 92% of samples and within-city source for 82% of samples. While soil and marine sampling sites were predicted for 86% and 74% of samples, respectively. Both studies by Danko et al. and Zhang et al. demonstrate the growing forensic value of environmental microbiomes as geolocation tools capable of linking biological material to specific locations.

#### Saliva microbiome

In a forensic study looking at the saliva microbiome, Liang et al. developed a random forest model from * 16S rRNA* data to distinguish saliva at the genus level between different geographical regions. The model exhibited some level of misclassification, as only 16 out of the 21 samples were correctly matched to one of the five geographic regions. Despite this, the study highlighted the potential for biogeographic inference and warrants more in-depth research with a larger sample size [62].

#### Skin microbiome

Similarly, skin samples can also provide information about geolocation, as demonstrated by Zeng et al. [63] differences in skin microbiomes have been observed in individuals residing in areas of high and low altitudes. The study was conducted in Tibet, and skin samples from 99 humans and 82 pigs were collected. Higher abundance of bacterial taxa (*Arthrobacter* sp., *Paenibacillus* sp., and *Carnobacterium* sp.) was present in samples from higher altitudes. There was a significantly lower difference (*P* < 0.01) in alpha diversity present in participants living at higher altitudes. In addition, many unique taxa associated with both humans and pigs that were discovered are known to be extremophiles, adapted to the harsh conditions found at high altitudes. These results may provide an avenue for determining geolocation based on altitude parameters [63]. However, future research will need to adjust methodologies appropriately and other changes within the skin microbiome, throughout an individual’s lifespan, will also need to be taken into consideration [64].

Lei et al.[65] investigated the potential of the human microbiome in predicting geolocation. A total of 220 swabs of the palmar skin, oral and nasal cavity were collected from individuals living in four different provinces in China: Shanghai, Chifeng, Kunming and Urumqi. PacBio sequencing of the * 16S rRNA* gene was used for microbiome analysis. Random forest algorithm demonstrated that bacteria from the palmar skin was the most appropriate for predicting geolocation compared to oral and nasal bacteria. Amongst individuals from Shanghai and Kunming, *Cutibacterium* was the most abundant bacterium with a relative abundance of 31% and 20% respectively. While *Psychrobacter* (13%) and *Psychrobacillu*s (14%) were the most abundant in Chifeng and Urumqi. In Shanghai and Kunming *Cutibacterium acnes*, *Staphylococcus caprae*, *Staphylococcus epidermidis* and *Streptococcus mitis* species occupied more than 30% of palmar bacteria. While *Psychrobacter sanguinis* (12%) and *Psychrobacillus psychrodurans* (14%) were predominantly present in Chifeng and Urumqi respectively. The differences in bacterial species detected are likely due to environmental factors and the lifestyles of individuals living in these regions. The palm was regarded as a more accurate body site for prediction of geolocation compared to other body sites and warrants further investigation for forensic applicability.

The establishment of the forensic microbiome database (FMD) by Singh et al. [66] allows for the geographical comparison of * 16S rRNA* microbiome data from human skin, vaginal fluid, saliva and stools from 35 countries and can further assist researchers with geolocation inference. Currently, the database has information on the skin microbiome from four countries (Belgium, Canada, Japan and United States) and from 15 different skin body sites. However, till date, no African countries are represented on the database [66].

## Discussion

The individuality and stability of the skin microbiota makes it ideal for forensic identification when traditional methods fall short, because skin microbes act as invisible evidence left behind in the environment and on objects [14]. Even among identical twins, differences are observed in the skin microbiota [72]. However, before the skin microbiome can be applied regularly to healthcare and forensics, it is necessary to establish effective study designs and standardise methods for collection, storage and extraction of skin samples typically because the skin is a low-biomass sample. Currently, collection and extraction methods differ across studies, which can affect the reproducibility of experiments. To mitigate the influence of contamination on results, experiments must include negative controls, extraction blanks, and mock community standards [73]. Selection of an appropriate platform for sequencing should also be considered, as reference annotation can influence the interpretation of sequencing results for diagnostic, therapeutic and forensic purposes [74]. In addition, while there are several reported skin microbiome studies that use amplicon sequencing and shotgun metagenomics, very little research has been done on long-read sequencing. Therefore, future studies should carry out an extensive comparison of all three sequencing techniques and establish which methods, whether used alone or in combination, will yield the most accurate and quick results for analysis of skin microbial communities. Another challenge related to forensics is selecting an optimal skin swabbing site for forensic identification that will allow for the generation of sufficient microbial DNA for data generation and analysis. Yu et al. swabbed the scalp of participants but microbial DNA recovery was low although still sufficient for community analysis [75]. If the skin microbiome is to be admissible in court for forensic identification, additional research is needed on improving microbial DNA recovery from different skin sites and assessing which locations yield the best possible results for human identification. Furthermore, although studies have assessed the stability of the skin microbiome [3, 46, 47], it is of utmost importance to rigorously test the timeframe between collecting a swab from an object in contact with the skin and determining if the microbiome recovered is usable and reliable. The microbial communities on the swab may differ from an individual’s true microbiome, potentially compromising accuracy in human identification [42]. For example, a recent study demonstrated through simulated experimental models of participants touching a knife, that some specific microbials are transferred from individuals to the object. However, the persistence of these microbial transfers varies greatly because only 8.28% of individual-specific microbials were detected after 4 h and 6.95% remained after 24 h. These findings demonstrate the need for more comprehensive research on the dynamics of bacterial transfer and persistence on objects. Furthermore, the interaction of environmental factors with objects must be considered as well as the presence of environmental bacteria that may colonize the object. Since environmental bacteria may compete with microbials deposited by individuals, thus diminishing its persistence [15].

As time progresses, microbiome studies are more frequently accompanied by machine learning models which assist in analyzing the large amount of microbial data generated [76]. At present Random Forest is the most widely used machine learning method. It performs well on classification and regression tasks and is suitable for small sample sizes which is ideal for experiments in the field of biology [77]. Machine learning models have been developed for matching a sample to skin microenvironment [9, 70], predicting gender [9, 78], identifying individuals [12], matching objects/surfaces to someone who has likely touched them [47] and determining geolocation [65]. However, based on the current literature, there are no reports of a successful model to predict the race/ethnicity of individuals. Phan et al. [78] attempted to do so by using *Alloiococcus* as a biomarker for ethnicity prediction however, the model had a low accuracy of 56%. Creating a model that can predict an individual’s race/ethnicity would be highly advantageous for investigators to narrow down potential suspects. Initially, this model may be specific for a particular country or region. To ensure high accuracy, large datasets would be required to train the model [79] and therefore may work best with a population-specific database with information on the skin microbiome. While the use of machine learning in forensics is encouraged, numerous ethical challenges warrant consideration, such as selecting appropriate algorithms, choosing analysis platforms, and determining application standards [76].

If we are to fully understand the human microbiome, global representation is required and underrepresented populations need to be studied to enrich databases. From a forensic perspective, FMD does not contain data of the skin microbiome from African countries [80]. Additionally, insight on how the environment and climates of different countries shape the skin microbiome will also aid in improving geolocation [23]. It is difficult to accurately pinpoint sample origin in regions with high levels of human mobility, such as popular cities that attract visitors with diverse genetic backgrounds, diets and lifestyles, as these often exhibit overlapping bacterial profiles [65]. To mitigate overlapping of microbial signatures, increased sampling sizes within regions may indicate small population-specific differences as well as collecting more metadata on population demographics and climate with regards to seasonal variation may alleviate overlaps. Future forensic research can incorporate the influence of skin diseases on the composition of the skin microbiome. The World Health Organization (WHO) estimated that skin conditions affect 1.8 billion people at any given time [81]. Acne, atopic dermatitis, psoriasis and chronic wounds are among the common skin diseases affecting individuals worldwide [82]. In forensic investigations bacteria associated with specific skin conditions may shed light for profiling characteristics of a person of interest and may form part of an additional tool to assist in human identification.

Most microbiome-based studies rely on sequencing the hypervariable regions of the * 16S rRNA* gene which generally limits identification to the genus level. This level of resolution may be insufficient to meet the need for more detailed microbial information required for forensic applications [51]. Although long read sequencing of the * 16S rRNA* gene allows for species level identification [83], additional research could include exploring alternative markers to the * 16S rRNA* gene that allow for species level identification. An example of such a gene is DNA gyrase subunit B (*gyrB*) that has been successfully used in the food industry to detect closely related bacterial species from food products. A comparison of sequencing *gyrB* and * 16S rRNA* regions V3-V4 was done, and bacterial species richness was more accurate when targeting *gyrB* in comparison to the * 16S rRNA* gene [84].

## Conclusion

In forensic sciences, the skin microbiome seems promising as an alternative method for individual identification, that can be implemented when traditional STR profiling is challenging. Global representation of the skin microbiome is essential to ensure each population group is represented, as literature has shown that variation in the skin microbiome exits among racial groups and individuals living in different locations. To ensure application in both healthcare and forensic science, standardised protocols must be developed from collection to analysis of data to maintain consistency and reproducibility of results.

## Data Availability

Not applicable.
